# Three-Dimensional Collagen Type I Matrix Up-Regulates Nuclear Isoforms of the Microtubule Associated Protein Tau Implicated in Resistance to Paclitaxel Therapy in Ovarian Carcinoma

**DOI:** 10.3390/ijms16023419

**Published:** 2015-02-04

**Authors:** Hilal Gurler, Yi Yu, Jacqueline Choi, Andre A. Kajdacsy-Balla, Maria V. Barbolina

**Affiliations:** 1 Department of Biopharmaceutical Sciences, University of Illinois at Chicago, 833 South Wood Street, Chicago, IL 60612, USA; E-Mails: hgurler@uic.edu (H.G.); Yi.Yu@cshs.org (Y.Y.); 2 Department of Pathology, University of Illinois at Chicago, 840 South Wood Street, Chicago, IL 60612, USA; E-Mails: jjchoi84@uic.edu (J.C.); ABalla@uic.edu (A.A.K.-B.)

**Keywords:** serous epithelial ovarian carcinoma, microtubule associated protein tau, paclitaxel, three-dimensional collagen matrix

## Abstract

Epithelial ovarian carcinoma is the deadliest gynecologic malignancy. One reason underlying treatment failure is resistance to paclitaxel. Expression of the microtubule associated protein tau has recently been proposed as a predictor of response to paclitaxel in ovarian carcinoma patients. Expression of tau was probed using immunohistochemistry in 312 specimens of primary, and 40 specimens of metastatic, ovarian carcinoma. Serous epithelial ovarian carcinoma cell line models were used to determine the expression of tau by Western blot and immunofluorescence staining. Subcellular fractionation and Western blot were employed to examine nuclear and cytoplasmic localization of tau. Gene silencing and clonogenic assays were used to evaluate paclitaxel response. Tau was expressed in 44% of all tested cases. Among the primary serous epithelial ovarian carcinoma cases, 46% were tau-positive. Among the metastatic serous epithelial ovarian carcinomas, 63% were tau-positive. Cell culture experiments demonstrated that tau was expressed in multiple isoforms. Three-dimensional collagen I matrix culture conditions resulted in up-regulation of tau protein. Silencing of tau with specific siRNAs in a combination with three-dimensional culture conditions led to a significant decrease of the clonogenic ability of cells treated with paclitaxel. The data suggest that reduction of tau expression may sensitize ovarian carcinoma to the paclitaxel treatment.

## 1. Introduction

Ovarian carcinoma is a spectrum of diseases of epithelial, stromal, and germ cell origins with an annual incidence of over 238,000 and 21,000 cases worldwide, and in the US, respectively [1]. The most predominant type is epithelial ovarian carcinoma that, based on its mutational profile and histopathological characteristics, is subdivided into high-grade serous, low-grade serous, endometrioid, mucinous, and clear cell histotypes [2,3,4]. High-grade serous ovarian carcinoma is the most common subtype and comprises about 70% of epithelial ovarian carcinoma; it is mostly sporadic and has no clearly established signs or reliable molecular markers of early disease. As a result, the majority of patients with high-grade serous ovarian carcinoma are diagnosed after the metastasis has spread. The presence of peritoneal metastasis significantly decreases patients’ survival. The currently used standard of care, which includes surgery and chemotherapy with a taxane and a platinum agent, is highly effective only for patients at an early stage of ovarian carcinoma (up to 90% 5-year survival) and not as efficient when metastasis has spread (less than 50% 5-year survival). A significant barrier to improvement of the overall survival of patients with metastatic ovarian carcinoma is acquired and intrinsic resistance to chemotherapy.

Clinical studies have demonstrated that weekly paclitaxel use in comparison to the every-3-weeks schedule in the treatment of recurrent, suboptimally debulked, or platinum-resistant ovarian cancers significantly improved the overall survival [5,6,7,8,9]. However, development of paclitaxel resistance remains a barrier to reaching complete remission. Expression of class I, III, and IV α- and β-tubulins and microtubule associated protein tau (MAPT, tau) has been associated with paclitaxel resistance in specimens of ovarian carcinoma [10,11]. Microtubules are composed of α and β subunits of tubulin arranged head-to-tail into protofilaments [12]. Microtubule dynamics change throughout the cell cycle, and assembly/disassembly of the microtubules is required for attachment and separation of sister chromatids during the cell division. Paclitaxel binds to β-tubulin on the inner surface of the microtubule, leading to its stabilization [13]. Tau can also bind to the inner surface of the microtubule, where paclitaxel binds, as well as to its outer surface [14], hence, it could compete with paclitaxel for binding to the microtubules.

Tau was first isolated from brain and its function has been extensively associated with neurodegenerative diseases and tauopathies [15,16,17,18,19]. Expression of six different molecular weight tau isoforms has been reported to date [20]. Tau has been detected in the extracellular space, cytoplasm, and nucleus; It plays a role in multiple processes, such as formation of extracellular neurofibrillary tangles, stabilization of microtubules, inhibition of apoptosis, protection of DNA, and nucleolar organization [21,22,23,24,25,26,27]. Expression of tau by cancer cells has been associated with response to paclitaxel treatment [28,29]. Studies of breast carcinoma suggested that tau expression in patients with complete response is significantly lower compared to those whose cancer recurred. Furthermore, low tau expression may be used as a marker to select patients who are likely to respond to paclitaxel therapy, while those with high tau expression may derive less benefit from this treatment [30,31,32,33]. Similarly, studies of gastric carcinoma demonstrated that tau expression levels can predict paclitaxel response [34,35,36]. More recently, studies of ovarian carcinoma suggested that tau may be a marker for paclitaxel sensitivity, and negative tau expression may serve as a prognostic factor and a predictor of paclitaxel/platinum response [11,37].

In this report, we examined expression of tau in specimens of primary and metastatic ovarian carcinoma. Human-derived cell models of ovarian carcinoma were used to probe tau expression and to characterize paclitaxel response when tau was silenced with siRNA.

## 2. Results and Discussion

Epithelial ovarian carcinoma is the deadliest gynecologic malignancy often diagnosed after metastasis has already occurred. If diagnosed before the cancer has spread beyond the ovary, ovarian carcinoma could be efficiently treated with surgery. However, metastases from ovarian carcinoma manifest themselves as countless lesions colonizing surfaces of the peritoneal organs and tissues; These lesions often defy complete surgical resection. Most of these metastatic lesions are susceptible to currently used chemotherapy, such as a combination of carboplatin and paclitaxel. Unfortunately, most of the cancers frequently recur shortly after treatment and eventually become nonresponsive to this therapy. Characterization of the molecular targets responsible for the intrinsic and acquired resistance to chemotherapy could lead to the development of novel treatment approaches, which could overcome chemotherapy resistance and, thus, would result in longer survival, or even complete cures.

### 2.1. Expression of Tau in Primary and Metastatic Ovarian Carcinoma Specimens

**Figure 1:**
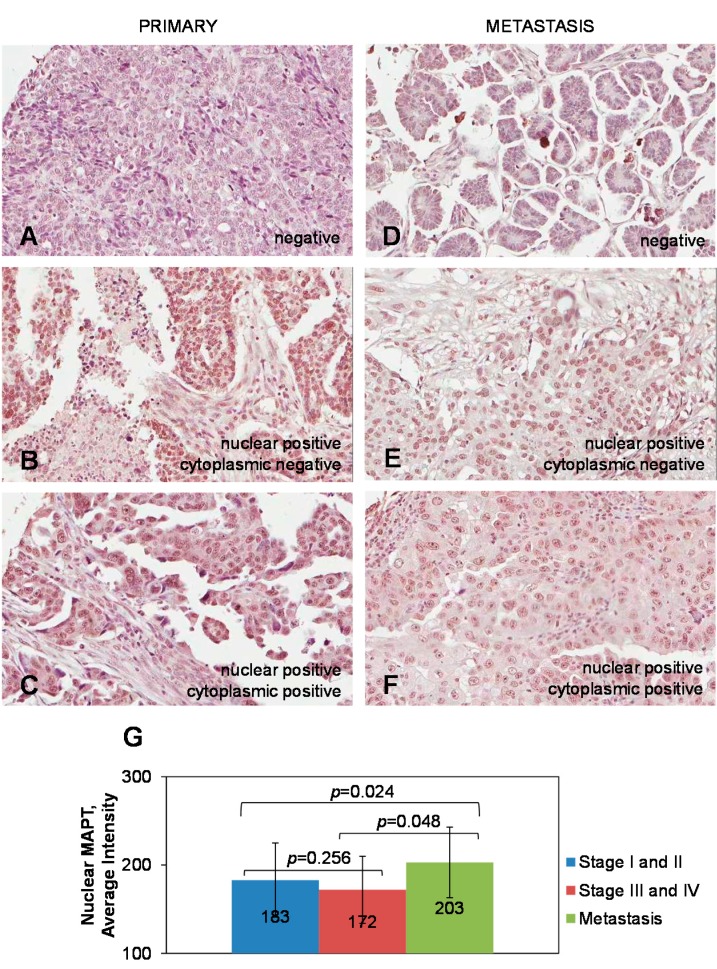
Expression of tau in specimens of primary and metastatic serous ovarian carcinoma. (**A**–**F**) Representative images of positive and negative tau expression in specimens of primary and metastatic serous ovarian carcinoma, as indicated. Brown color demonstrates tau staining. Blue color demonstrates staining with hematoxylin. Images were generated using an Aperio ScanScope digital slide scanner. Magnification: 10×; (**G**) Averaged tau expression in the specimens of primary and metastatic serous ovarian carcinoma. Nuclear intensity of tau ± standard deviation is shown. Data analysed with the Student’s *t*-test.

Expression of tau in ovarian carcinoma (stage I–IV) was proposed as a potential predictive marker of response to paclitaxel/platinum-based chemotherapy in ovarian carcinoma [11]. Because paclitaxel-resistant ovarian carcinoma is often characterized by advanced stage, high grade, and vast peritoneal metastasis, we hypothesized that tau could also be expressed in the metastatic specimens of ovarian carcinoma. Hence, we wished to determine tau expression in metastatic ovarian carcinoma specimens and compare it to that in the primary tissues. We used TMAs containing 312 specimens of primary and 40 metastatic ovarian carcinoma specimens, of which 181 primary and 38 metastatic specimens belonged to serous epithelial ovarian carcinoma, and tested tau expression by immunohistochemistry using tau antibody (5C6) that detects all isoforms of phosphorylated and nonphosphorylated tau. Detailed description of the tested cases and tau scores are presented in Tables S1–S3. Tested cases of primary ovarian carcinoma belonged to serous (*n* = 181), mucinous (*n* = 42), endometrioid (*n* = 19) epithelial ovarian carcinoma, and other epithelial, stromal, and germ cell ovarian tumors (*n* = 70). Tested cases of metastatic ovarian carcinoma disseminated to the omentum (*n* = 32) and other peritoneal tissues (*n* = 8) were of serous epithelial histotype (*n* = 38) and other epithelial origins (*n* = 2). Specimens with score < 100 were considered tau-negative, and those with scores >100, tau-positive. We have found that tau expression was positive in 44% of all tested cases and its expression localized mostly to the cell nuclei (Figure 1A–F). Overall average score for all tested primary ovarian carcinomas was 96, and the overall average score for all tested metastatic ovarian carcinoma specimens significantly increased to 137 (*p* = 0.0088; Student’s *t*-test). Among the primary serous epithelial ovarian carcinoma cases, 46% were tau-positive. Among the metastatic serous epithelial ovarian carcinomas 63% were tau-positive. For the primary tau-positive serous ovarian carcinoma specimens average score of tau expression was 182. The average score for metastatic tau-positive serous carcinoma specimens was 203, and was significantly higher compared to that for the primary tau-positive serous carcinomas (*p* = 0.018; Student’s *t*-test). Among the tau-positive primary serous ovarian carcinomas, the average score for Stage I and II specimens did not significantly change compared to that for Stage III and IV specimens. However, changes in the average score for tau-positive metastatic serous ovarian carcinoma specimens were significantly higher compared to those for either Stage I and II or Stage III and IV tau-positive serous ovarian carcinoma specimens (Figure 1G). These data suggest existence of mechanisms whereby tau is up-regulated in ovarian carcinoma metastasis.

Previous studies have indicated that tau is expressed in primary ovarian carcinomas and could be used as a predictor of paclitaxel therapy response in ovarian carcinoma. Our data suggests an increase in the number of tau-positive cases in metastatic specimens compared to that for the specimens of primary ovarian carcinoma. Furthermore, analysis of our data revealed an increase of an average intensity of tau staining in metastatic specimens compared to that in the primary ovarian carcinoma. These data may suggest that an increase of tau expression in metastatic ovarian carcinoma could be one of the factors behind failure of paclitaxel treatment in this patient cohort.

### 2.2. Expression of Tau in Serous Epithelial Ovarian Carcinoma Cell Culture Models

**Figure 2:**
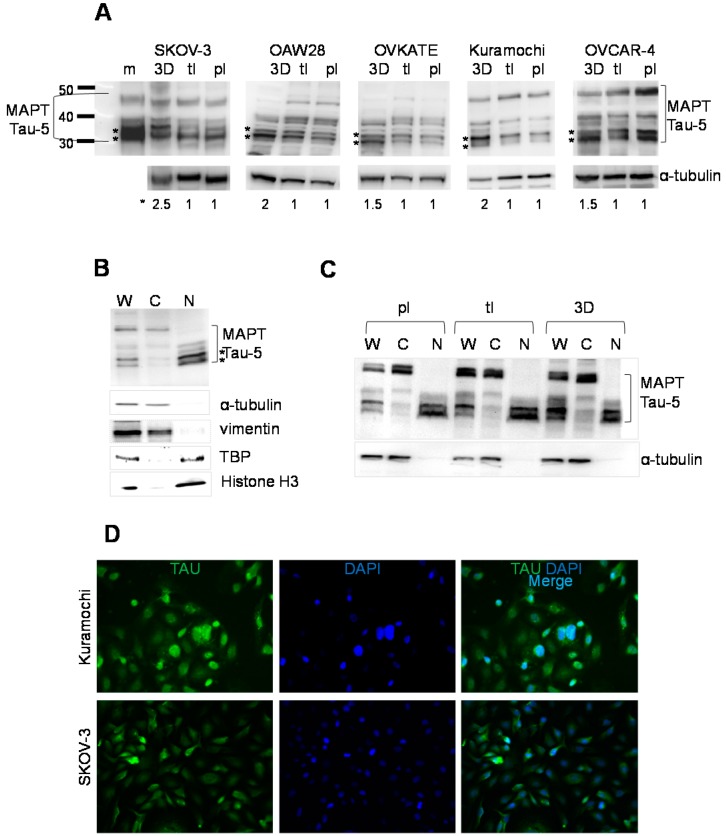
Expression of tau in cell culture models of serous ovarian carcinoma. (**A**) Expression of tau (**top panels**) in the cell culture models of ovarian carcinoma, as indicated, cultured in the indicated conditions was determined using Western blot. Positive control for tau “m” was used to determine positioning of the tau isoforms. Two lower molecular weight tau isoforms up-regulated when cells were cultured in three-dimensional collagen type I matrix (3DCIM) conditions are indicated with stars. α-Tubulin (**bottom panels**) was used as a loading control and to calculate normalized intensity of two lower molecular weight isoforms of tau (at the bottom of the gel images) using digital densitometry; ** indicate positions of the lower molecular weight tau isoforms; * indicates normalized tau as quantified by dividing the intensity corresponding to all tau isoforms by that corresponding to α-tubulin. (**B**) A representative Western blot image showing tau localization in the whole cell lysate (W), as well as its cytoplasmic (C) and nuclear (N) fractions of the tested serous ovarian carcinoma cell lines cultured on 3DCIM, as described in the Methods. Asterisks show positions of two lower molecular weight tau isoforms. α-Tubulin and vimentin were used as loading control for the cytoplasmic protein fraction, and tata binding protein (TBP) and histone H3 were used as loading controls for the nuclear protein fraction; (**C**) A representative Western blot image showing tau localization in the whole cell lysate (W), as well as its cytoplasmic (C) and nuclear (N) fractions of the tested serous ovarian carcinoma cell lines cultured on plastic supports (pl), type I (tl), and 3DCIM, as described in the Methods. α-Tubulin was used as loading control for the cytoplasmic protein fraction; (**D**) Immunofluorescence images of tau expression in serous ovarian carcinoma cell lines, as indicated. Images were generated using a Zeiss fluorescence microscope and 20× objective lens. The fluorescence signal from tau staining (green) was overlaid with DAPI staining (blue) to generate a merged image (light blue).

It is thought that specific microenvironments at the metastatic sites can affect tumor cells and support the development of more aggressive and treatment-resistant phenotypes. We have previously demonstrated that three-dimensional collagen type I matrix (3DCIM) culture conditions, mimicking extracellular matrix in the peritoneal cavity, can be used as a model to study interaction of metastasizing ovarian carcinoma cells with the extracellular matrix in the microenvironment of metastatic ovarian carcinoma [38,39,40,41,42,43]. Hence, we tested expression of tau in serous ovarian carcinoma cell lines SKOV-3, OAW28, OVKATE, Kuramochi, and OVCAR4 cultured atop 3DCIM, and those plated on thin-layer coated collagen type I (tl) tissue culture treated plastic supports (pl). Genomic profiling studies have demonstrated that these cell lines belong to the serous histotype of epithelial ovarian carcinoma; moreover, all but SKOV-3 belong to the high-grade subtype of the disease [44]. After 24 h of culture, cells were collected as detailed in Methods and their total cell lysates were used to test expression of tau with the Tau-5 clone of tau antibody that detects all tau isoforms regardless of their phosphorylation status. Our data demonstrate that multiple tau isoforms were expressed in all tested cell lines (Figure 2A,B). Interestingly, 3DCIM culture conditions generated significantly stronger signal associated with two lower molecular weight tau isoforms in all tested cell lines (Figure 2A). Because our immunohistochemistry data demonstrated that tau was localized mostly to the nucleus of the tested human ovarian carcinoma specimens, we used cell lines cultured atop 3DCIM in subcellular fractionation experiments and probed nuclear and cytoplasmic lysates with tau antibody using Western blot. These experiments demonstrate that the two bands corresponding to the lower molecular weight tau isoforms predominantly localize to the nucleus (Figure 2B). Subcellular fractionation of cells cultured either on tl or pl resulted in the same pattern of nuclear and cytoplasmic distribution of tau isoforms (Figure 2C). Immunofluorescence staining with tau (5C6) antibodies also confirmed strong nuclear tau expression in cell culture models of serous epithelial ovarian carcinoma (Figure 2D). Of note, in the conditions when cell membranes were permeabilized with 0.1% Triton X-100, as detailed in Methods, a distinct nuclear pattern of tau was seen in all tested cell culture models. However, omission of cell permeabilization (*i.e.*, no addition of Triton X-100) from the immunofluorescence staining protocol produced only cytoplasmic tau staining (Figure S1), suggesting that visualization of tau staining strongly depends on the specific conditions of the detection method used. Altogether, these data may indicate that collagen type I matrix in the peritoneal microenvironment could be one of the factors that up-regulates expression of nuclear tau isoforms.

### 2.3. Silencing of Tau with Specific siRNAs Results in Paclitaxel Sensitization in Cells Cultured Atop Three-Dimensional Collagen Type I Matrix (3DCIM)

**Figure 3:**
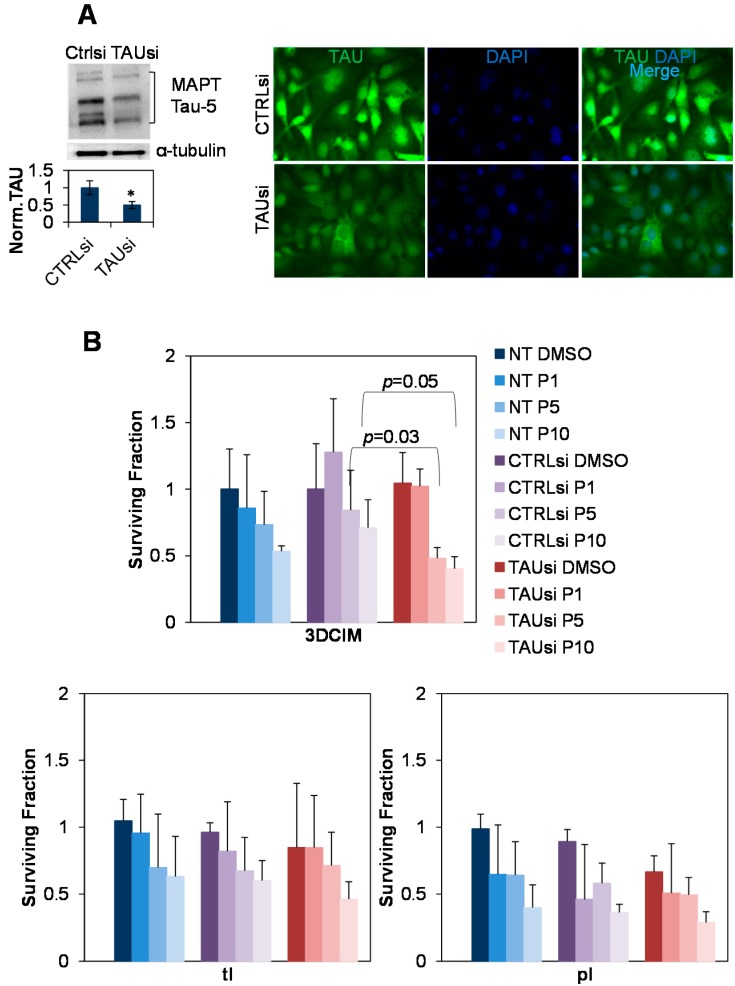
Down-regulation of tau reduces clonogenic ability of SKOV-3 cultured atop three-dimensional collagen type I matrix. (**A**) Cells were transiently transfected with either control (CTRLsi) or tau-specific (TAUsi) siRNAs for three days followed by detection of Tau expression using Western blot and immunofluorescence staining. Down-regulation of tau was quantified using Western blot and digital densitometry (**right panel**). Normalized tau was quantified by dividing intensity values corresponding to all tau isoforms by those corresponding to α-tubulin. α-Tubulin was used as a loading control. *, *p* < 0.05; Student’s *t*-test. Loss of both nuclear and cytoplasmic tau after silencing with tau-specific siRNAs was visualized using immunofluorescence staining with anti-tau antibodies (**left panel**). Images were generated using a Zeiss fluorescence microscope and 20× objective lens. The fluorescence signal from tau staining (green) was overlaid with DAPI staining (blue) to generate a merged image; (**B**) Cells were transiently transfected with either control (CTRLsi) or Tau-specific (TAUsi) siRNA, or non-transfected (NT) for three days, then replated atop three-dimensional collagen type I matrix (3DCIM), thin lay er coated collagen type I (tl), and plastic supports (pl), treated with paclitaxel at 1, 5, and 10 nM (designated P1, P5, and P10, respectively) or DMSO as a control for 24 h followed by clonogenic assay as indicated in the Methods.

**Figure 4:**
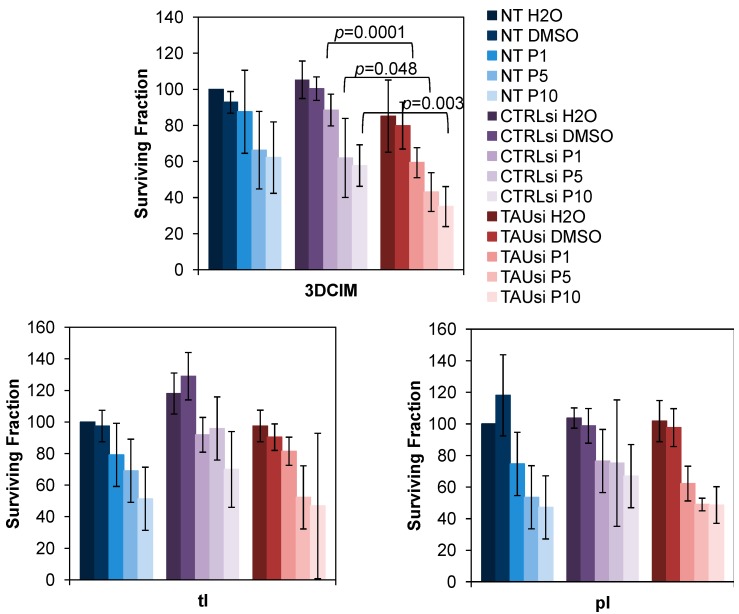
Down-regulation of tau reduces cell survival of SKOV-3 cultured atop three-dimensional collagen type I matrix. Cells were transiently transfected with either control (CTRLsi) or Tau-specific (TAUsi) siRNA, or non-transfected (NT) for three days, then replated atop three-dimensional collagen type I matrix (3DCIM), thin layer coated collagen type I (tl), and plastic supports (pl), treated with paclitaxel at 1, 5, and 10 nM (designated P1, P5, and P10, respectively), as well as H_2_O or DMSO as a controls for 24 h. Cells that survived the treatment were collected and counted. Cell numbers collected from non-transfected H20-treated controls (NT H_2_O) were arbitrarily set as “100”, and cell numbers for all other conditions were recalculated and plotted accordingly.

As previous correlative studies associated expression of tau with resistance to paclitaxel in ovarian carcinoma, we tested paclitaxel response in SKOV-3 cell culture model. Tau was silenced with specific siRNAs and the cells were cultured atop 3DCIM. Efficiency of tau down-regulation in our experiments was approximately 50% as demonstrated by Western blot and immunofluorescence staining (Figure 3A). Our data demonstrate that down-regulation of tau with specific siRNAs results in a significant reduction of the clonogenic ability of paclitaxel-treated cells cultured atop 3DCIM (Figure 3B, top panel). In contrast, in cells cultured on tl and pl, reduction of tau expression did not induce statistically significant reduction of clonogenic ability of paclitaxel-treated cells (Figure 3B, bottom panels). Additionally, down-regulation of tau with specific siRNAs led to a significant reduction of the cell survival of paclitaxel-treated cells cultured atop 3DCIM (Figure 4). These data may suggest that nuclear tau isoforms that are up-regulated by the 3DCIM culture are playing a role in paclitaxel resistance in ovarian carcinoma, and, when silenced, lead to sensitization to paclitaxel treatment.

The microenvironment at metastatic sites could affect gene expression leading to the development of drug resistance. Our data indicate that three-dimensional collagen type I culture conditions, modeling peritoneal extracellular matrix, result in up-regulation of tau, suggesting that interaction with the extracellular matrix at the metastatic sites could lead to up-regulation of tau. Interestingly, the specific tau isoforms up-regulated by the three-dimensional collagen type I matrix were localized to the cell nucleus. Nuclear tau, which has been previously reported in both normal and cancer cells, has been implicated in the inhibition of apoptosis, DNA protection, and nucleolar organization, but the role of nuclear tau in paclitaxel response has not been tested. In our experiments, silencing of tau resulted in significant reduction of the clonogenic ability when cells were cultured atop three-dimensional collagen matrix, but not the planar substrata. These observations suggest that the lower molecular weight tau isoforms expressed by ovarian carcinoma cells compete with paclitaxel for binding to the microtubules, thus, attenuating the efficacy of the drug. This is consistent with previous conclusions regarding the roles of individual tau isoforms in different neuronal pathologies [45]. Thus, our data suggest that tau expression in ovarian carcinoma has a functional role in regulating paclitaxel response.

In light of the observation that the tau protein responsible for paclitaxel resistance resides in the nucleus, it is interesting to speculate regarding a possible spatio-temporal mechanism of tau-dependent paclitaxel resistance. It is known that paclitaxel stabilizes microtubules and stops the cell cycle in G2/M phase. Formation of the mitotic spindle and attachment of the chromosomes takes place after the nuclear envelope breaks down to release the DNA. Liberated nuclear tau could bind to the microtubules and, thus affect binding of paclitaxel, making it less efficient. As the efficacy of paclitaxel treatment could have major ramifications for the patients with ovarian carcinoma, both mechanisms of tau expression and competition with paclitaxel to microtubule binding warrant future investigation.

## 3. Experimental Section

### 3.1. Materials

Tissue microarray (TMA) slides OV2161, OV2087, and OV808 containing human specimens of normal and pathologic ovarian tissue, as well as metastatic ovarian carcinoma, were obtained from US Biomax (Rockville, MD, USA). Vectastain ABC and 3,3'-diaminobenzidine (DAB) HRP substrate kits were obtained from Vector Laboratories (Burlingame, CA, USA). Rat tail collagen type I was obtained from BD Biosciences (Bedford, MA, USA). Human tau (pool of 3 proprietary 19–25 nt sequences) and control siRNAs, as well as a mouse anti-vimentin antibody were obtained from Santa Cruz Biotechnology (Santa Cruz, CA, USA). Mouse anti-TATA binding protein (TBP) antibody, rabbit anti-histone H3 antibodies, and anti-mouse biotin-conjugated antibodies were obtained from Abcam (Cambridge, MA, USA). Mouse anti-tau (Tau-5 clone) antibody, a positive control for anti-tau (Tau-5 clone) antibody, and DharmaFECT1 were obtained from Fisher Scientific (Pittsburg, PA, USA). Mouse anti-tau antibody (5C6) and mouse anti-human-α-tubulin were obtained from Iowa Developmental Studies Hybridoma Bank (Iowa City, Iowa, IA, USA). Paclitaxel was obtained from Sigma–Aldrich (St. Louis, MO, USA).

### 3.2. Cell Lines

Human ovarian carcinoma cell lines of serous histotype originating from malignant cells in ascites, OVCAR-4 and SKOV-3, were obtained from the NCI Tumor Cell Repository (Detrick, MD, USA). These cell lines were cultured as suggested by the manufacturer. Human ovarian carcinoma cell lines of serous histotype OAW28 as well as Kuramochi, OVSAHO, and OVKATE were obtained from Sigma–Aldrich (St. Louis, MO, USA) and the Japanese Collection of Research Bioresources Cell Bank (Osaka, Japan), respectively, and were cultured as suggested by the manufacturers. All cell lines were routinely assessed for cellular morphology and average doubling time. All cell lines were propagated from stocks originally obtained from cell banks and individual investigators and have been stored in aliquots for future use. Each aliquot was further propagated for no longer than twenty consecutive passages or four months, whichever came first.

### 3.3. Immunohistochemistry

Procedures were performed as we described previously [43,46,47]. Briefly, TMA slides were rehydrated by incubation in xylene (twice, 10 min each), 100% ethanol (twice, 5 min each), 95% ethanol (twice, 3 min each), 80% ethanol (2 min), 70% ethanol (2 min), and phosphate buffered saline, pH 7.4, (5 min). Peroxidase activity was blocked with H_2_O_2_. Antigen retrieval was achieved with a 10-min microwaving in 10 mM sodium citrate, pH 6.0. Prior to primary antibody staining (mouse monoclonal Tau 5A6 antibodies from the Developmental Studies Hybridoma Bank at 6 μg/mL overnight at 4 °C), non-specific binding was blocked by incubation with 10% goat serum for 1 h. Biotin-conjugated goat anti-mouse secondary antibody was used at a dilution of 1:200 for 30 min at room temperature (RT). Vectashield ABC was used as suggested by the manufacturer and incubated with tissues for 45 min at RT. DAB reagent was prepared as suggested by the manufacturer, applied to the tissues on the TMA slide for up to 10 min until a brown color developed. TMAs were stained with hematoxylin, dehydrated in 50%, 70%, 95%, 100% ethanol, and xylene and mounted with Permount. Specimens of normal brain were used as a positive control, and exclusion of primary antibody was used as a negative control. Staining was evaluated by Andre A. Kajdacsy-Balla and Jacqueline Choi, who were both blinded to the experimental outcomes of the study. Two scoring parameters were used: intensity of staining and percentage of positive cells. Staining was scored based on the intensity and percentage of positive cells. Intensity of staining was “0” for negative cases, “1” for weakly positive cases, “2” for moderately positive cases, and “3” for highly positive cases. Overall scores were derived as the intensity score multiplied by the percentage of positive cells, as previously described [48]. Staining was assessed separately in the cytoplasm and in the nucleus.

### 3.4. Immunofluorescence Staining

The cells were cultured on glass coverslips, fixed, permeabilized with 0.1% Triton X-100 (when needed), and blocked in 10% goat serum for 1 h at RT. Mouse anti-human-tau (5A6) antibodies were used at a 1:100 dilution and incubated with cells for 2 h at RT. Secondary anti-mouse Alexa433-conjugated antibodies were used at 1:500 and incubated with cells for 1 h at RT in the dark. DAPI (4',6-Diamidino-2-phenylindole) was added to the secondary antibody solution to a final concentration of 10 μg/mL 10 min prior to the end of the incubation period. The cells were washed, air dried, and mounted on glass slides using ProlongGold (Invitrogen, Carlsbad, CA, USA). Fluorescent imaging was performed using a Zeiss AxioObserverD.1 fluorescence microscope.

### 3.5. Subcellular Fractionation

Subcellular fractionation to separate nuclear and cytoplasmic fractions of the cell lysates was performed as previously described [43]. Briefly, cells were cultured on tissue culture treated plastic supports (designated “pl”), thin-layer coated collagen type I (10 μg/mL; designated “tl”), and three-dimensional collagen type I matrix (0.8 mg/mL; designated “3DCIM”) for 24 h, released with 0.05% trypsin-EDTA or 10 μg/mL collagenase, respectively, and collected by centrifugation for 5 min at 6000 rpm at 4 °C. Cells were resuspended in 50 mM TRIS, pH 8.0, containing 150 mM NaCl and 1% Nonidet P40, incubated on ice for 5 min, spun down for 5 min at 12,000 rpm, and supernatant containing cytoplasmic fraction was removed. The pelleted nuclei were resuspended in 50 mM HEPES, pH 7.5, containing 150 mM NaCl, 10 mM EDTA, 1% Triton X-100, 0.5% Sodium Deoxycholate, and 0.1% SDS, and incubated 2 min on ice, briefly vortexed, returned to ice, and the procedure was repeated four times, then centrifuged as above, and supernatants containing nuclear fractions were removed. Protein concentration was measured with Bradford protein assay, and processed for Western blots. All cell lysis buffers contained Protease inhibitor cocktail (Roche, Indianapolis, IN, USA).

### 3.6. Western Blot

Western blotting analysis was used to detect the expression of tau and α-tubulin in cell culture models of ovarian carcinoma. This procedure was performed as previously described [38,39,41]. Briefly, antibodies were used at the following dilutions: 1:200 mouse anti-human-tau (Tau-5, Thermo Scientific, Waltham, MA, USA) in 3% BSA in deionized water for 2 h at RT, and 1:500 mouse anti-human-α-tubulin in 3% BSA in deionized water for 1 h at RT. Immunoreactive bands were visualized with an anti-(mouse-IgG)-peroxidase (Sigma, St. Louis, MO, USA) (1:1000 in 3% BSA in a solution of 50 mM tris-buffered saline, pH 7.4, 150 mM NaCl, and 0.05% Tween-20 (TBST), and enhanced chemiluminescence was read using Chemidoc (Bio-Rad, Hercules, CA, USA) and Bio-Rad Chemidoc ImageReader software (Bio-Rad).

### 3.7. Transient Transfections

Ovarian carcinoma cells were cultured to 80% confluence and transfected with siRNAs using DharmaFECT1 according to the manufacturer’s instructions.

### 3.8. Paclitaxel Treatment, Cell Survival, and Clonogenic Assay

Cells were treated with 1, 5, and 10 nM paclitaxel dissolved in DMSO for 24 h. Control conditions included cells treated with H_2_O and DMSO. Cells cultured on plastic supports and thin-layer collagen type I were collected by trypsinization. Cells cultured atop 3DCIM were liberated with collagenase as described before [39]. Live cells were counted. One percent of the collected cells were plated to assess their clonogenic ability. The cells were cultured in standard conditions for ten days followed by fixing the colonies with 4% paraformaldehyde. Colonies were stained with 0.05% crystal violet solution in water for 30 min, and the dye was extracted by adding methanol to the stained cells. OD_540_ measurements were taken as a measure of colony formation and plotted on the graphs as “surviving fraction”. Counting colonies instead of taking OD_540_ measurements was also performed and produced similar results in the differences in cell survival.

## 4. Conclusions

Our data indicate up-regulation of tau expression in the specimens of metastatic ovarian carcinoma in comparison to those of the primary site. Cell culture experiments demonstrate that collagen matrix could be one of the factors regulating tau by inducing expression of its isoforms located in the nucleus. Furthermore, silencing of tau leads to sensitization to paclitaxel in cells cultured atop the collagen matrix. These studies indicate that tau has a functional role in regulating the response to paclitaxel treatment in ovarian carcinoma, and support the need for further investigations to understand the role tau plays in chemoresistance of ovarian carcinoma.

## Supplementary Materials

Supplementary materials can be found at http://www.mdpi.com/1422-0067/16/02/3419/s1.
